# Nitrate Prevents Sjögren's Disease by Modulating T Helper Cells via NF‐κB Pathway Suppression

**DOI:** 10.1111/odi.70004

**Published:** 2025-06-16

**Authors:** Conglin Du, Zi Yang, Yang Yang, Chunmei Zhang, Hideaki Kagami, Xianqi Li, Liang Hu, Junji Xu, Jian Zhou

**Affiliations:** ^1^ Salivary Gland Disease Center and Beijing Key Laboratory of Tooth Regeneration and Function Reconstruction, Beijing Laboratory of Oral Health and Beijing Stomatological Hospital Capital Medical University Beijing China; ^2^ Department of Endodontics, Beijing Stomatological Hospital Capital Medical University Beijing China; ^3^ Department of Oral and Maxillofacial & Head and Neck Oncology Capital Medical University School of Stomatology, Beijing Stomatological Hospital, Capital Medical University Beijing China; ^4^ Department of Dentistry and Oral and Surgery Aichi Medical University Nagakute Aichi Japan; ^5^ Department of Oral and Maxillofacial Surgery, School of Dentistry Matsumoto Dental University Shiojiri Japan; ^6^ Department of Oral and Maxillofacial Surgery Capital Medical University School of Stomatology Beijing China; ^7^ Department of Periodontology Stomatological Hospital of Chongqing Medical University Chongqing China; ^8^ Department of VIP Dental Service, Beijing Stomatological Hospital Capital Medical University Beijing China; ^9^ Laboratory for Oral and General Health Integration and Translation, Beijing Tiantan Hospital Capital Medical University Beijing China

**Keywords:** autoimmune disease, nitrate, salivary glands, secondary Sjögren's disease, T helper cells

## Abstract

**Objective:**

Sjögren's disease (SjD) is a chronic autoimmune disease characterized by abnormal T helper (Th) cell distribution in the salivary glands (SGs). Although nitrate can regulate immune responses and preserve SGs function, its preventive effects on SjD remain unexplored.

**Methods:**

Nonobese diabetic (NOD)/Ltj mice were used as a secondary Sjögren's disease (sSjD) animal model. From 6 to 16 weeks of age, the mice received either sodium nitrate (2 mM) in drinking water (SjD + Nit group) or sodium chloride (SjD + NaCl group). Salivary flow rates were recorded biweekly, and submandibular glands were collected at 16 weeks for histological analysis. Th subsets ratios were determined using flow cytometry, and mRNA sequencing was used to explore nitrate's preventive mechanism.

**Results:**

Nitrate pretreatment preserved salivary function and reduced lymphocyte infiltration. T cell related genes were downregulated, while salivary function genes were upregulated. Differentiation pathways for Th cell subsets were downregulated in the SjD + Nit group, and nitrate modulated the abnormal balance of Th cells, regulatory T cells (Tregs) in the SGs, and peripheral blood, suppressing the NF‐κb pathway of sSjD.

**Conclusions:**

Preventive nitrate administration may preserve SG function, rebalance Th cells, and suppress NF‐κB pathway, offering a potential treatment for SjD.

## 1 Introduction

Sjögren's disease (SjD) is a chronic systemic autoimmune disease primarily affecting the exocrine glands, particularly the salivary and lacrimal glands, resulting in dry mouth and eyes. The global incidence of SjD is approximately 6 per 100,000, with a tenfold higher prevalence in women (Qin et al. 2015). However, no effective clinical methods are currently available for preventing SjD progression.

The primary pathogenesis of SjD is complex and not yet fully elucidated. The salivary glands (SGs) of patients with SjD are characterized by the infiltration of immune cells, especially T and B cells (Zhan et al. 2023). Among these, CD4^+^ T cells are predominant in SGs infiltrates and can polarize into different lineages, including T helper (Th) 1, Th17, and regulatory T cells (Tregs) after activation (Christodoulou et al. 2010). These polarizations contribute to the severity of SjD by producing pro‐inflammatory cytokines, including IFN‐γ, which induce B cell activation and differentiation, promote the formation of ectopic germinal centers, and facilitate the initiation and progression of SjD (Verstappen et al. 2018, 2021; Xu et al. 2022). Increasing evidence indicates that the overexpression of Th17 cells and reduced Treg cell numbers may drive SjD development (Iizuka et al. 2015). Thus, targeting Th1, Th17, and Tregs is a promising strategy for developing immunotherapies for SjD (Psianou et al. 2018).

Nitrate, primarily obtained through green leafy vegetables, is crucial in maintaining hemostasis during systemic health (Qu et al. 2016; Ma et al. 2018). Nitrate supplementation has beneficial effects on endothelial function, modulates inflammation, reduces oxidative stress, and protects against ischemia reperfusion (Weitzberg and Lundberg 2013; Chang et al. 2019; Wang et al. 2020; Li, An, et al. 2021; Li, Jin, et al. 2021). Nitrate is concentrated and reabsorbed by SGs (Qin et al. 2012), and nitrate deficiency is associated with SG dysfunction (Xia et al. 2003a, 2003b). They can improve the functional activity of SGs in estrogen deficiency‐induced xerostomia (Xu et al. 2018) and prevent irradiation‐induced SG damage (Feng et al. 2021; Li, An, et al. 2021; Li, Jin, et al. 2021; Pan, Gu, et al. 2023; Pan, Hu, et al. 2023), suggesting that dietary nitrate may prevent hyposalivation. Additionally, nitrate can modulate the immune response, alleviate dextran sulfate sodium (DSS)‐induced colitis by inhibiting interleukin (IL)‐17 expression (Hu et al. 2020), and prevent the progression of bisphosphonate‐related osteonecrosis of the jaw (BRONJ) by inhibiting monocyte necroptosis (Pan, Gu, et al. 2023; Pan, Hu, et al. 2023). However, the potential benefits of preventive nitrate administration for SjD remain unexplored.

This study used nonobese diabetic (NOD)/Ltj female mice as a model for secondary Sjögren's disease (sSjD) – a form of autoimmune exocrinopathy that typically arises secondary to systemic autoimmune disorders such as rheumatoid arthritis or systemic lupus erythematosus, characterized by lymphocytic infiltration of salivary glands and progressive loss of secretory function (Sebastian et al. 2019). Sodium nitrate was administered to NOD/Ltj mice via drinking water from 6 to 16 weeks old to evaluate the preventive effect of nitrate on sSjD. We analyzed SG function following nitrate supplementation and investigated the underlying mechanisms of interactions between nitrate and the immune system in sSjD.

## 2 Materials and Methods

### 2.1 Animals

Forty‐two 4‐week‐old female NOD/Ltj mice were kept under standardized conditions at 21°C–22°C with 12‐h light/dark cycles. The animals had free access to water and regular pellet food for 7 days before the experiment. The animal study was conducted in accordance with the Animal Care and Use Committee of the Capital Medical University (Code: AEEI‐2017‐116).

Twenty‐four mice were then randomly divided into three groups: SjD (NOD/Ltj mice received normal water, *n* = 8), SjD + Nit (NOD/Ltj mice were treated with 2 mM sodium nitrate (NaNO_3_), *n* = 8), and SjD + NaCl (NOD/Ltj mice were treated with 2 mM sodium chloride (NaCl), *n* = 8). In the SjD + Nit and SjD + NaCl groups, NaNO3 or NaCl was administered in drinking water at a concentration of 2 mM from 6 to 16 weeks of age. Mice of the three groups were sacrificed at 16 weeks of age, and submandibular glands, blood, and urine samples were collected for further experiments. Other eighteen NOD/Ltj mice received normal water. Mice were sacrificed, and samples were collected at weeks 6, 8, 10, 12, 14, and 16 to detect the changes during the progression of NOD/Ltj mice (*n* = 3 per time point).

### 2.2 Salivary Flow Rate

Saliva flow rates for all mice were recorded at weeks 6, 8, 10, 12, 14, and 16. The animals were anesthetized with pentobarbital sodium (50 mg/kg). Stimulated saliva flow was measured 10 min after an intraperitoneal injection of pilocarpine (50 mg/mL) at a dose of 0.1 mL/kg body weight. Animals were placed in the lateral position. Cotton balls were placed under the tongue and held steadily during a 10‐min period to collect saliva. The weight difference of the cotton balls was calculated.

### 2.3 Nitrate Levels in the Serum, Saliva, Urine and SG Tissue

Blood samples were collected after animals were anesthetized, and serum was obtained by centrifugation at 1500 rpm for 30 min at room temperature. Saliva and urine were centrifuged at 3000 rpm for 30 min at 4°C. A sample of the submandibular gland (100 mg) was homogenized in 200 μL of reaction dilution buffer (892877, R&D, MN, United States) and centrifuged at 12,000 rpm for 15 min. The supernatant was then collected for further analysis. Saliva, urine, serum, and tissue supernatants were filtered by a 10‐kDa Ultrafiltration Tube (Millipore, USA) and centrifuged at 12,800 × *g* for 20 min. The total nitric oxide and nitrate/nitrite parameter assay kit (KGE001, R&D, MN, United States) was used to determine the concentration of nitrate.

### 2.4 Histological Analysis

At week 16, submandibular glands were collected. Part of submandibular glands were fixed in 4% paraformaldehyde for histological analysis. Fixed samples were embedded in paraffin and sliced into 5 μm sections. Hematoxylin and eosin staining of the SG was performed to observe inflammatory infiltration. The counts and areas of inflammatory foci containing > 50 lymphocytes per 4 mm^2^ of tissue were calculated. Infiltrating areas (%) were quantified using image pro plus 6.0 by threshold‐based segmentation of hematoxylin‐stained regions. Three nonoverlapping fields per gland were analyzed.

### 2.5 Immunofluorescent (IF) Staining

All samples were fixed simultaneously with 4% paraformaldehyde for 24 h, and embedded in paraffin and sliced into 5 μm sections. Paraffin sections were subjected to IF staining after gradient alcohol dehydration. After antigen retrieval with citrate antigen retrieval solution (BL619A; biosharp, Anhui, China), nonspecific protein adsorption was blocked using 5% bovine serum albumin (BSA; Beyotime, Shanghai, China). The sections were incubated overnight with primary antibodies against AQP5 (1:500, ab78486; Abcam, Waltham, MA, USA), T‐bet (1:500, 97135S, Cell Signaling Technology), and RORγt (1:3000, ab207082, Abcam) at 4°C and subsequently incubated with secondary antibodies (1:1000, F‐2765, Thermo Fisher, Waltham, MA, USA) and Fluoroshield with 4′,6‐diamidino‐2‐phenylindole (DAPI) (Sigma‐Aldrich Corp., St. Louis, MO, USA). The stained samples were evaluated under a fluorescence microscope (BX61; Olympus, Tokyo, Japan) with the same illumination parameters. AQP5 staining Integrated Optical Density (IOD) and tissue area were quantified with Image Pro Plus 6.0 software after background subtraction via region of interest (ROI) analysis. The number of T‐bet and RORγt‐positive cells within the tissue was determined using the same software.

### 2.6 Immunohistochemical (IHC) Staining

After antigen retrieval and blocking, sections were incubated overnight with primary antibodies against the following: p‐P65 (1:1000, 3031; Cell Signaling Technology, Danvers, MA, USA), Foxp3 (1:1000, ab215206, Abcam) and Gata3 (1:500, 5852S, Cell Signaling Technology) at 4°C and subsequently incubated with secondary antibodies (PV‐8000, ZSGB‐BIO, Beijing, China). The sections were then stained using a SignalStain DAB Substrate Kit (8059S, Cell Signaling Technology) and evaluated under a microscope (BX61, Olympus). The number of p‐P65, Foxp3, and Gata3 positive cells within the tissue was determined by Image Pro Plus 6.0.

### 2.7 Flow Cytometry

Peripheral blood samples were collected and transferred to tubes containing sodium heparin. First, 1X red blood cell lysis buffer (R1010, Solarbio, Beijing, China) was added, and peripheral blood mononuclear cells were centrifuged at 1000 rpm. Cells were blocked with anti‐CD16/32 (1:200, 14‐0161‐82, Thermo) for 10 min before staining. Antibodies against the following were used for flow cytometry analysis: CD4 (1:100, FITC, RM4.5, BD Biosciences, San Jose, CA, USA), CD25 (1:100, APC, PC61, BD Biosciences), IFN‐γ (1:100, PE‐Cy7, XMG1.2, BD Biosciences), IL‐4 (1:100, APC, 11B11, BD Biosciences), IL‐17 (1:100, PE, TC11‐18H10, BD Biosciences), and FOXP3 (1:100, PE; 259D/C7, BD Biosciences).

The SGs were digested with collagenase type II (4 mg/mL, Worthington Biochemical, Lakewood, NJ, USA) and dispase type II (4.67 mg/mL, Worthington Biochemical) combined in Hanks' balanced salt solution for 15 min at 37°C. A single‐cell suspension was blocked with anti‐CD16/32 (1:200, 14‐0161‐82, Thermo) for 10 min, then stained with the following antibodies: CD4 (1:100, 100559, BV510, RM4‐5, Biolegend San Diego, CA, USA) and CD45 (1:100, 147716, Alexa Fluor 700, I3/2.3, Biolegend). For intracellular cytokine staining, cells were stimulated with Cell Stimulation Cocktail (00‐4970‐93, Invitrogen, Waltham, MA, USA) and protein transport inhibitor cocktail (00‐4980‐93, Invitrogen) at 37°C for 6 h, followed by fixation with the Fixation/Permeabilization buffer solution (554714, BD Bioscience), then stained with the following antibodies: IL‐17A (1:100, 506922, PE‐Cyanine7, TC11‐18H10.1, Biolegend) and IFN‐γ (1:100, 505817, Pacific Blue, XMG1.2, Biolegend). Foxp3/Transcription Factor Fixation/Permeabilization Concentrate and Diluent (00‐5521, Invitrogen) was used for intranuclear staining and the following antibodies were used: FOXP3 (1:100, 126,409, Pacific Blue, MF‐14, Biolegend), GATA3 (1:100, APC, 653805, Biolegend), T‐bet (1:100, PE‐Cyanine7, 25‐5825‐82, Thermo), and RORγt (1:100, PerCP‐eFluor 710, 46‐6981‐80, Thermo). Stained cells were analyzed using an LSRFortessa (BD Biosciences), and data were analyzed using FlowJo software (Tree Star, Ashland, OR, USA).

### 2.8 Serum Inflammatory Multifactorial Cytokine Detection

Serum was collected and stored at −80°C. Multiple mouse Th cytokines were tested using the LEGENDplex Mouse Th Cytokine Panel (13‐plex) (740005; BioLegend). The reaction mixture of each test comprised: 25 μL assay buffer, 25 μL sample, 25 μL beads, and 25 μL antibody. Flow cytometry was performed using a LSRFortessa and analyzed using the FlowJo software. The concentrations of transforming growth factor‐beta 1 (TGF‐β1) in serum were measured using an enzyme‐linked immunosorbent assay (ELISA) kit (1217102, Mouse TGF‐β1 Precoated ELISA Kit; Dakewe, Shenzhen, China). Serum samples were diluted and activated with 1 N HCl for 30 min at room temperature (RT), followed by neutralization with 1.2 N NaOH and Dilution Buffer R (1×). After biotinylated antibody and streptavidin‐HRP were sequentially applied, each followed by incubation and washing steps, TMB substrate was then added and incubated at 37°C for 20 min before stopping the reaction. Absorbance was measured at 450 nm.

### 2.9 RNA Sequencing

Submandibular glands were randomly selected from the SjD group (*n* = 3) and SjD + Nit group (*n* = 3) for RNA sequencing. A comparison was performed to identify genes that were differentially regulated between the SjD and SjD + Nit groups. The preparation of transcriptome libraries and sequencing were performed by OE Biotech Co. Ltd. (Shanghai, China). A *p*‐value < 0.05 was set as the threshold for significant differential expression. Reactome enrichment analysis and Gene Set Enrichment Analysis (GSEA) were performed using R based on a hypergeometric distribution. The datasets analyzed in the present study are available in the National Center for Biotechnology Information (NCBI) with the primary accession code PRJNA1161122.

### 2.10 Western Blotting (WB) Analysis

The samples obtained from animals were prepared for WB analysis. Total protein was extracted with lysis buffer (C1053, Applygen, Beijing, China) and quantified using a BCA protein assay kit (GK10009; GlpBio, Montclair, CA, USA). Equal amounts of protein extracts were loaded onto FuturePAGE 4%–20% polyacrylamide gels (ACE, Nanjing, China) for electrophoresis and then transferred to nitrocellulose membranes. The membranes were blocked with 5% nonfat dry milk, followed by incubation at 4°C overnight with the primary antibodies against P65 (1:1000, 3034; Cell Signaling Technology), p‐P65 (1:1000, 3033; Cell Signaling Technology), IκBα (1:1000, 9242; Cell Signaling Technology), p‐IκBα (1:1000, 2859S, Cell Signaling Technology), and β‐actin (1:20000, AC026; Abclonal, Wuhan, China). This was followed by incubation with secondary antibodies. The immunoblot bands were visualized using an enhanced chemiluminescent detection system (ChemiDoc MP Imaging System, Bio‐Rad, Hercules, CA, USA).

### 2.11 Quantitative Real‐Time Polymerase Chain Reaction (qRT‐PCR)

Total RNA was obtained from submandibular glands of different groups using the RNAprep pure Tissue Kit (DP41, Tiangen, Beijing, China). Then, RNA was reverse‐transcribed into cDNA using the NovoScript Plus All‐in‐one 1st Strand cDNA Synthesis SuperMix (E047‐01A, Novoprotei). qRT‐PCR was performed using the NovoStartSYBR qPCR SuperMix Plus (E096‐01B, Novoprotein). The primers tested were listed in Table S1.

### 2.12 Statistical Analysis

Data were analyzed using SPSS 22.0, and charts were produced using GraphPad 9.0. The one‐way ANOVA test was used to determine significant differences among multiple groups. Student's *t*‐test was performed to compare differences between two groups with a normal distribution. Data are shown as mean ± standard error (M ± SEM), and significance is presented as * (*p* < 0.05), ** (*p* < 0.01), and *** (*p* < 0.001).

## 3 Results

### 3.1 Nitrate Supplement Prevents Progression of SG Dysfunction in NOD/Ltj Mice

The NOD/Ltj mouse is a spontaneous secondary SjD model characterized by lymphocyte infiltration in the submandibular glands and a significantly reduced salivary flow rate. The area of lymphocyte infiltration increased from 6 to 16 weeks in NOD/Ltj mice (*r* = 0.9914, *p* < 0.001; Figure S1A,B). A decrease in salivary flow rate was observed from week 8, with levels maintained at a low rate after week 10 (*r* = −0.8963, *p* < 0.05; Figure S1C). Similarly, the salivary nitrate concentration began to decline at week 10 and leveled off at week 14 (*r* = −0.9073, *p* < 0.05; Figure S1D). These results indicate that the early stage of the sSjD model was established at week 10 and decrease in nitrate was significantly associated with the development of sSjD.

**FIGURE 1:**
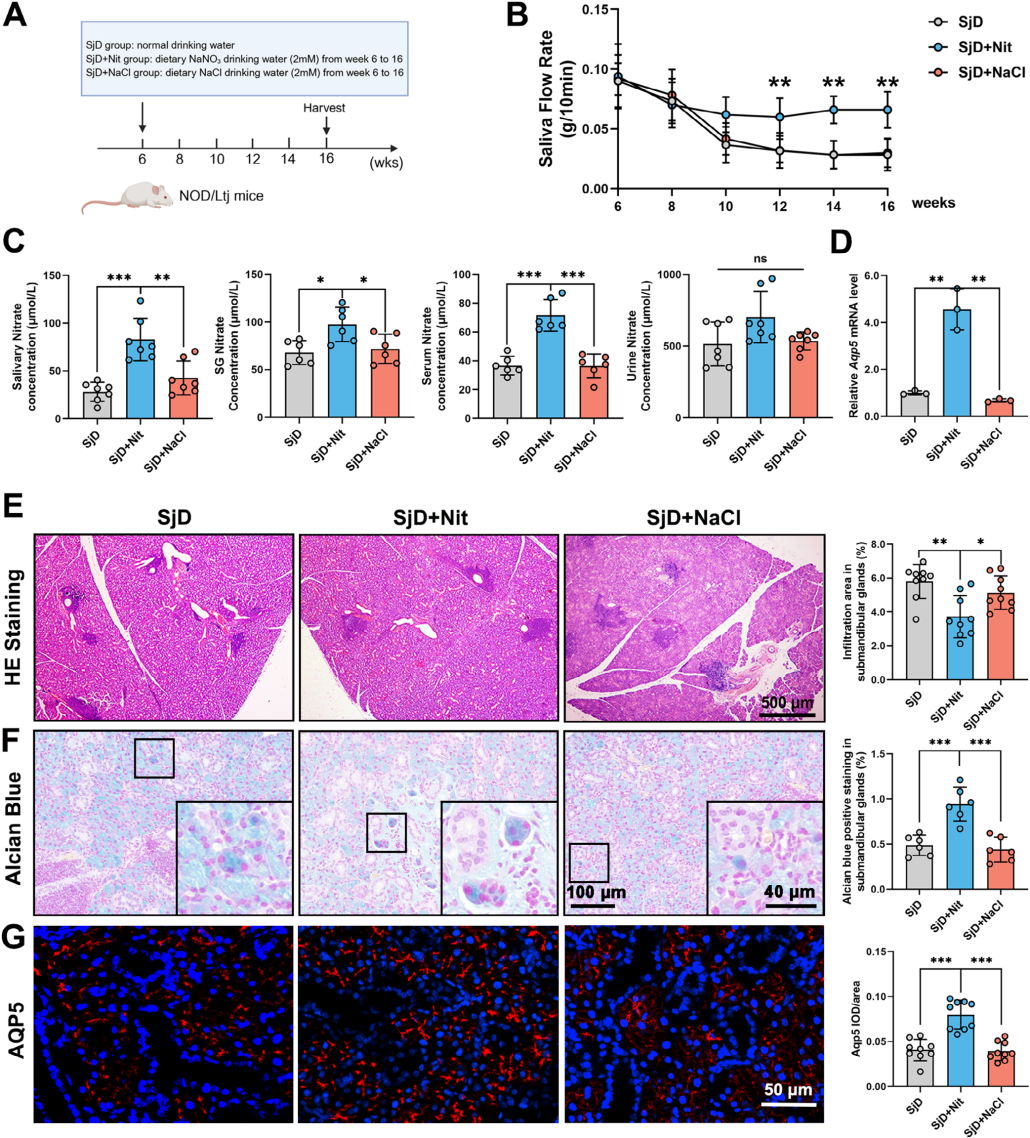
Effect of nitrate on preventing Sjögren's disease in NOD/LtJ mice. (A) Schematic of the animal experiment and treatment. NOD/LtJ mice were treated with NaNO_3_ and NaCl from week 6 to 16. Samples were collected at 16 weeks. (B) The salivary flow rate (g/10 min) in NOD/LtJ mice was significantly reduced at week 10 and remained low and significantly recovered in SjD + Nit group (*n* = 8, **p < 0.01). (C) Change of nitrate concentration in NOD/LtJ mice. The nitrate concentration of SjD + Nit group increased in the saliva, submandibular glands and serum. In the urine, no significant difference was found among three groups (*n* = 6–7, ^ns^no significant difference, *p < 0.05, **p < 0.01, ***p < 0.001). (D) Change of mRNA expression of *Aqp5* (*n* = 3, **p < 0.01). (E) HE‐stained histological images of submandibular glands and quantitative analysis of infiltration area showed that nitrate could significantly reduce the lymphocyte infiltration in submandibular glands of NOD/LtJ mice. (*n* = 9, scale bars: 500 μm; *p < 0.05, **p < 0.01). (F) Alcian blue staining showed that nitrate could increase sulfated oligosaccharides in salivary glands (*n* = 6, ***p < 0.001). (G) Immunofluorescence staining of AQP5 (red) was significantly increased in the SjD + Nit group compared with the SjD and SjD + NaCl groups (*n* = 9, scale bar: 100 μm, ***p < 0.001). ANOVA test was used to determine significant differences among multiple groups.

To assess the preventive effect of nitrate, drinking water containing nitrate or NaCl was supplied from week 6. After 10 weeks of treatment, salivary function and histological examinations were performed at week 16. The experiments were conducted as shown in Figure 1A. Salivary flow rate recovered from week 10 and significantly increased at weeks 12, 14, and 16 after nitrate supplementation (**p < 0.01; Figure 1B). In the SjD + Nit group, nitrate concentration in saliva was significantly higher than in the SjD and SjD + NaCl groups (**p < 0.01, ***p < 0.001 Figure 1C). Changes in nitrate levels in serum and submandibular glands were consistent with those in saliva (*p < 0.05, ***p < 0.001; Figure 1C). No significant differences were observed in nitrate concentrations in the urine (ns = *p* > 0.05, Figure 1C), indicating that more nitrate enters the bloodstream and is reabsorbed in the SjD + Nit group.

As indicated by hematoxylin and eosin (HE) staining, the number and area of lymphocyte infiltration foci in the SjD + Nit group were significantly lower than those in the SjD and SjD + NaCl groups (*p < 0.05, **p < 0.01; Figure 1E). Alcian blue staining positive area increased in SjD + Nit group compare with SjD group and SjD + NaCl group, indicating nitrate increased sulfated oligosaccharides in acinar cells (***p < 0.001; Figure 1F). Aquaporin‐5 (AQP5), a member of the transmembrane aquaporin family that contributes to transepithelial water movement, is crucial in saliva secretion (Xu et al. 2022). In the SjD + Nit group, integrated optical density normalized to tissue area (IOD/Area) of Aqp5 (red staining) in the membrane of acinar cells was increased and *Aqp5* expression in SjD + Nit group increased (**p < 0.01, ***p < 0.001; Figure 1D,G), indicating that salivary flow was maintained through nitrate prevention. Overall, preventive nitrate supplementation inhibited lymphocyte infiltration and revitalized salivary flow rate in sSjD.

### 3.2 Nitrate Prevention Is Associated With Th Cell‐Related Gene Expression and NF‐κB Signaling Pathway in NOD/Ltj Mice

**FIGURE 2:**
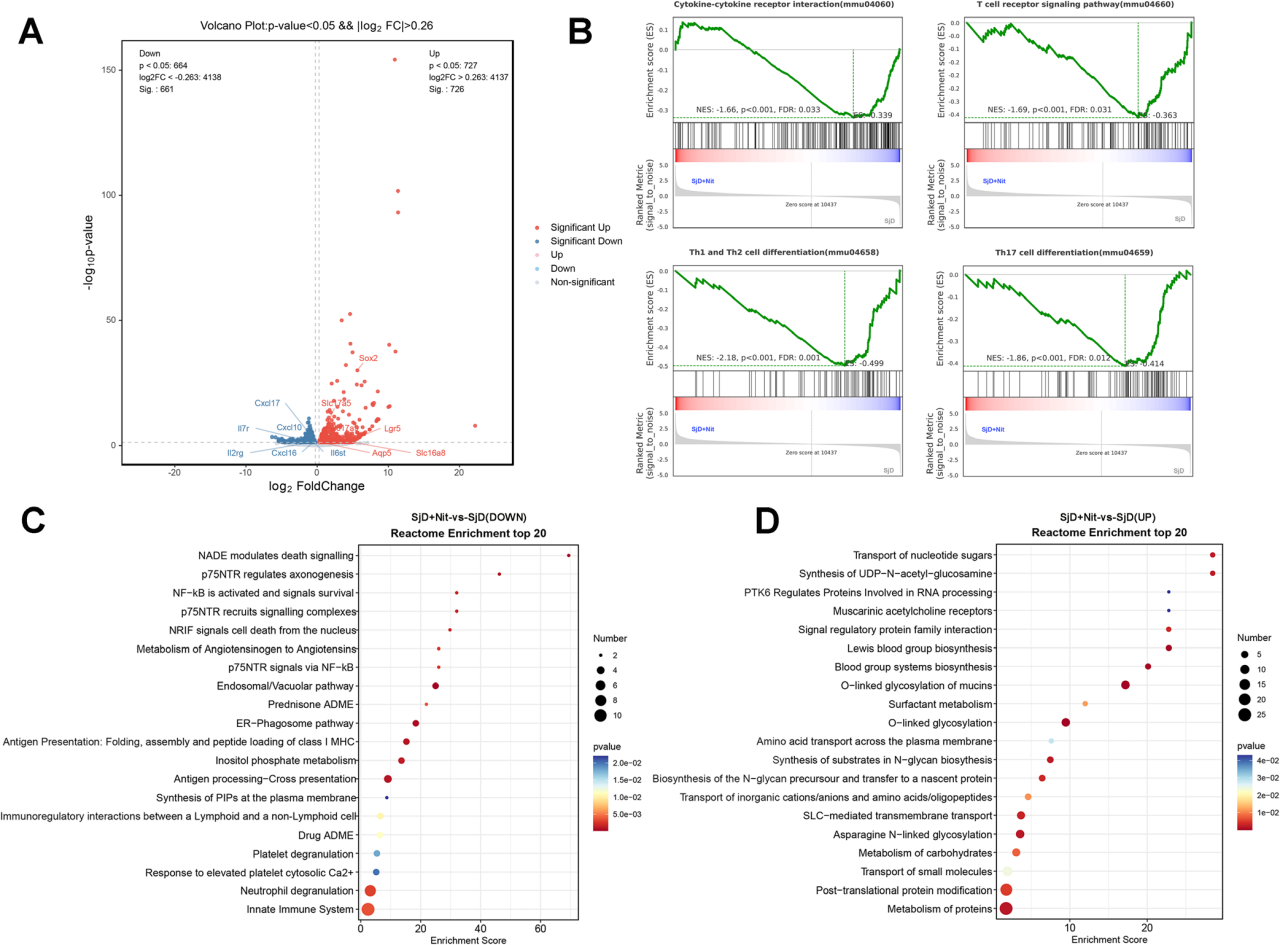
RNA sequencing of submandibular glands in SjD and SjD + Nit group (*n* = 3). (A) Volcano plot of differentially regulated genes. (B) GSEA showed that cytokine‐cytokine receptor interaction, Th17 cell differentiation, Th1 and Th2 cell differentiation, T cell receptor signaling pathway were significantly downregulated in the SjD + Nit group. (C, D) Top 20 of reactome enrichment of SjD and SjD + Nit groups.

To further explore the underlying molecular mechanisms, we performed RNA sequencing of SjD and SjD + Nit groups. A total of 1391 differentially expressed genes (DGEs) were identified in the SjD + Nit group compared with the SjD group, with 727 upregulated and 664 downregulated. Compared with the SjD group, genes related to salivary secretory function—*Aqp5, Sox2*, and *Lgr5*—were upregulated in the SjD + Nit group. Genes related to cytokines and chemokines, such as *Il7r, Il2rg, Il6st, Cxcl10*, and *Cxcl16*, were downregulated (*p* < 0.05, Figure 2A). *Il7r, Il2rg, Il6st, Tlr2, Tlr4*, and *Myd88* were downregulated in the SjD + Nit group at the mRNA level (**p < 0.01, ***p < 0.001; Figure S2A). The expression of the nitrate‐related transport gene *Slc17a5* significantly increased after nitrate administration (*p* < 0.05; Figure S2B). Genes related to salivary glands *Muc5b* and *Amy1* were upregulated at the mRNA level (*p < 0.05, **p < 0.01; Figure S2C). GSEA showed that cytokine‐cytokine receptor interaction, Th17 cell differentiation, Th1 and Th2 cell differentiation, and the T cell receptor signaling pathway were downregulated in the SjD + Nit group (*p* < 0.05, Figure 2B). Nitrate upregulated *Foxp3*, *Gata3*, and downregulated *Rorc*, *Tbx21* at the mRNA level (**p < 0.01, ***p < 0.001; Figure S2D) suggesting that nitrate potentially prevents the inflammation of sSjD and is associated with modulating Th cell differentiation. Additionally, nitrate upregulated *Nfkbib, Ngf*, *Ngfr*, and *Sort1*, and downregulated *Ikbkg*, *Rela*, and *ntrk1* at the mRNA level (*p < 0.05, **p < 0.01, ***p < 0.001; Figure 5G, Figure S2E). Nitrate supplementation downregulated the innate immune system, neutrophil degranulation, and p75 neurotrophin receptor (p75NTR) signals and nuclear factor kappa‐light‐chain‐enhancer of activated B cells (NF‐κB) (*p* < 0.05; Figure 2C). Reactome enrichment analysis showed nitrate supplementation is associated with the transport of small molecules and the solute carrier (SLC)‐mediated transmembrane transport pathway compared with the SjD group (*p* < 0.05; Figure 2D), indicating that preventive nitrate administration enhanced the transport function of sSjD. Transcriptional profiling revealed that nitrate pretreatment was associated with the upregulation of pathways linked to secretory processes, such as mucin O‐glycosylation and posttranslational protein modifications (*p* < 0.05; Figure 2D). Besides, *St6galnac2, B3gnt6, Ogt, Dpagt1, Stt3a*, and *Stt3b* mRNA levels were upregulated in the SjD + Nit group, suggesting a potential role of nitrate in modulating salivary gland secretion (*p < 0.05, **p < 0.01; Figure S2F). Genes related to chemokines *Cxcl2*, *Cxcl1*, *Ccl2*, and *Ccl5* were downregulated in the SjD + Nit group at the mRNA level (*p < 0.05, **p < 0.01; Figure S2G). Genes related to ER stress *Atf6, Ern1, Ddit3*, and *Xbp1* were downregulated in the SjD + Nit group at the mRNA level (*p < 0.05, ***p < 0.001; Figure S2H). Overall, nitrate was linked to altered expression of genes related to SG transport function, chemokine signaling, Th‐cell differentiation, and NF‐κB pathways.

### 3.3 Inorganic Nitrate Maintains Th Lymphocyte Balance in SGs and Peripheral Blood

**FIGURE 3:**
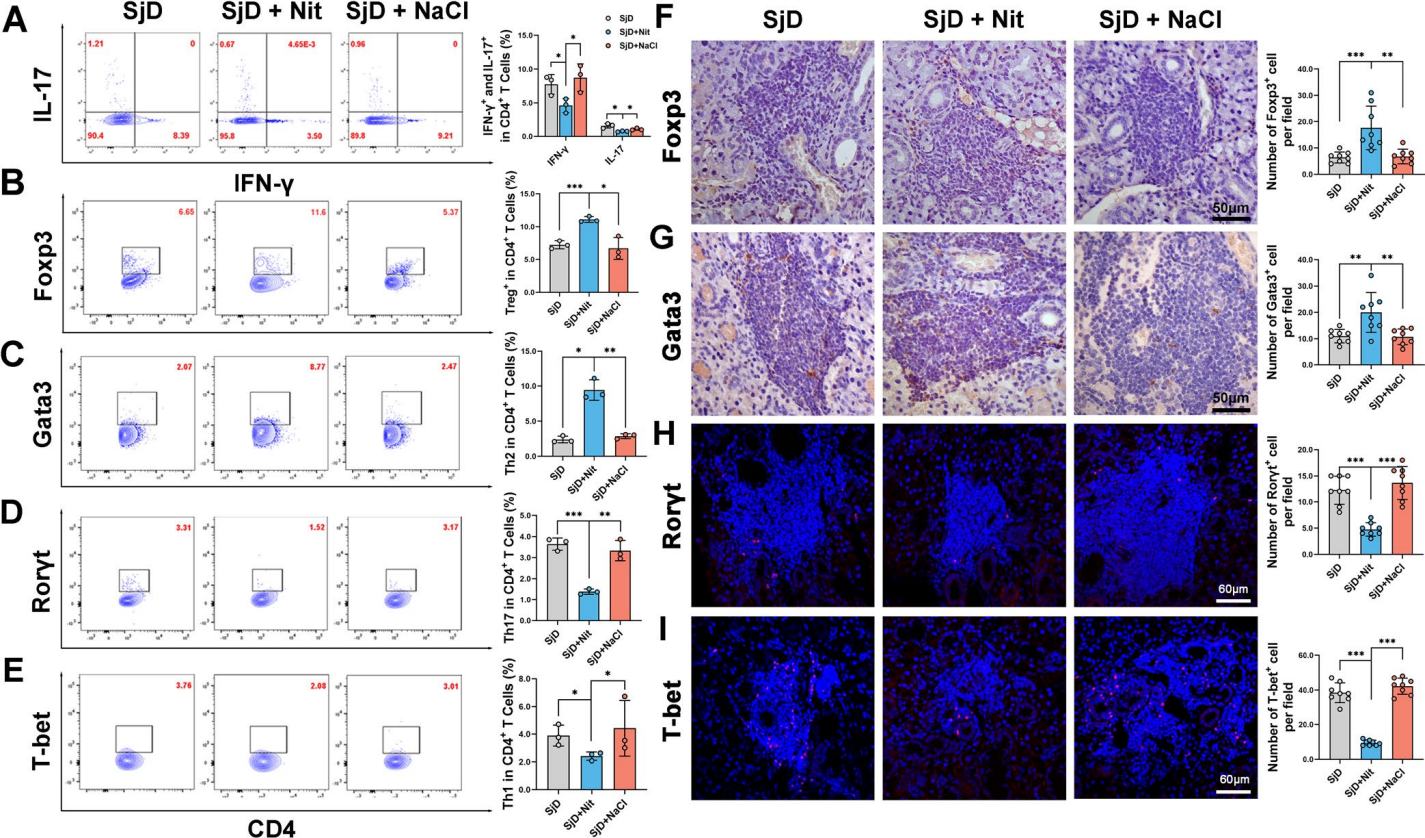
Nitrate could modulate the imbalance of Th lymphocyte in submandibular glands of NOD/LtJ mice. (A–E) Nitrate could up‐regulate ratio of Treg cells and Th2 cells and downregulate ratio of Th1 and Th17 cells in submandibular glands of NOD/LtJ mice (*n* = 3, *p < 0.05, **p < 0.01, ***p < 0.001). (F–I). IHC staining and IF staining showed that the expressions of T‐bet and RoRγt in submandibular glands of NOD/LtJ mice was significantly decreased, while GATA3 and FOXP3 were significantly increased in the SjD + Nit group (*n* = 8, scale bar: 50 μm and 120 μm, **p < 0.01, ***p < 0.001). ANOVA test was used to determine significant differences among multiple groups.

An imbalance in Th cells contributes to sSjD pathogenesis through the production of pro‐inflammatory cytokines. The ratios of Th lymphocyte subsets were determined using flow cytometry. The results showed that CD4^+^/IFN‐γ^+^ or CD4^+^/IL17^+^ cells, representing Th1 or Th17 cells, respectively, were significantly lower in the submandibular glands of the SjD + Nit group than in the SjD group (*p < 0.05; Figure 3A). The ratios of Tregs (CD4^+^/Foxp3^+^) and Th2 (CD4^+^/Gata3^+^) were significantly higher in the SjD + Nit group than that in the SjD and SjD + NaCl group (*p < 0.05, **p < 0.01, ***p < 0.001; Figure 3B,C). Additionally, Th1 (CD4^+^/T‐bet^+^ cells) and Th17 (CD4^+^/Rorγt^+^ cells) also decreased in SjD + Nit group compared with SjD and SjD + NaCl group (*p < 0.05, **p < 0.01, ***p < 0.001; Figure 3D,E). The expression of T‐bet, GATA3, RoRγt, and FOXP3 in submandibular glands were also assessed via IHC staining and IF staining. The positive staining of T‐bet and RoRγt were significantly decreased, while GATA3 and FOXP3 were significantly increased in the SjD + Nit group (**p < 0.01, ***p < 0.001; Figure 3F–I). Overall, the ratio of Th1 and Th17 cells decreased and the ratio of Th2 and Treg cells increased in the SjD + Nit group compared with the SjD group, indicating that preventive nitrate treatment maintained the Th lymphocyte cell balance in the SG, which may underlie its preventive effects on sSjD.

**FIGURE 4:**
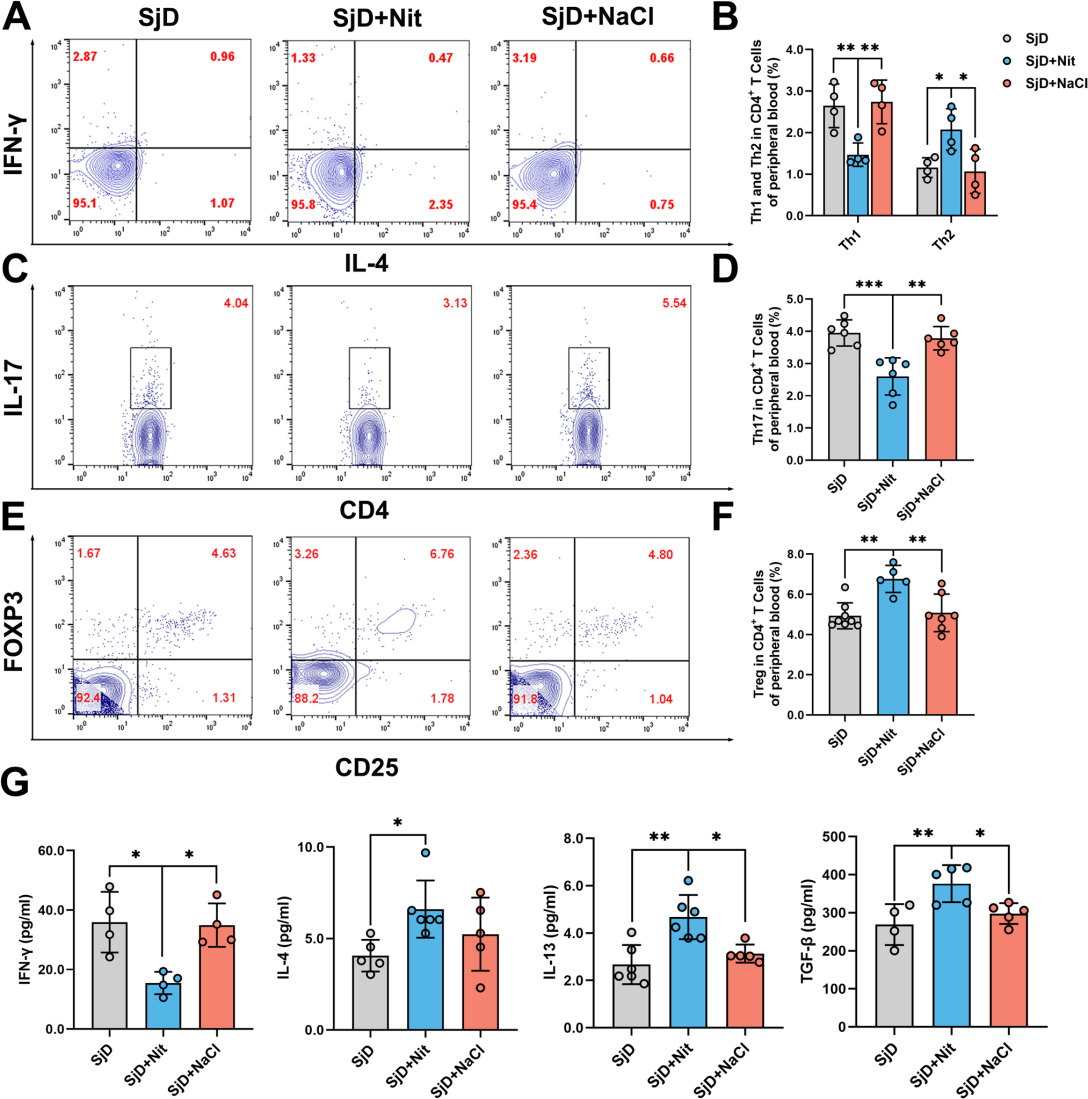
Nitrate could regulate the imbalance of Th lymphocyte cells and cytokines in peripheral blood of NOD/LtJ mice. (A–F) Nitrate could up‐regulate Treg and Th2 cells and downregulate Th1 and Th17 cells in peripheral blood of NOD/LtJ mice (*n* = 4–8, *p < 0.05, **p < 0.01, ***p < 0.001). (G) Pro‐inflammatory cytokines (IFN‐γ) were downregulated and the anti‐inflammatory cytokines (IL‐4, IL‐13, and TGF‐β) were upregulated in the SjD + Nit group (*n* = 4–6, *p < 0.05, **p < 0.01). ANOVA test was used to determine significant differences among multiple groups.

Subsequently, we examined changes in Th lymphocyte subsets in the peripheral blood and cytokines in the serum. We observed that nitrate moderated the balance between peripheral blood Th17/Treg and Th1/Th2 cells in NOD/LtJ mice (*p < 0.05, **p < 0.01, ***p < 0.001; Figure 4A–F). Meanwhile, IFN‐γ, an essential pro‐inflammatory cytokine, was significantly decreased, while the anti‐inflammatory cytokines, including IL‐4, IL‐13, and TGF‐β1, were increased in the SjD + Nit group compared with the SjD group (*p < 0.05, **p < 0.01; Figure 4G). Overall, nitrate inhibited inflammation and cytokine production and re‐established the systemic and submandibular gland T cell balance.

### 3.4 Nitrate‐Induced Improvement in SG Function Is Associated With Suppression of the NF‐κB Pathway

**FIGURE 5:**
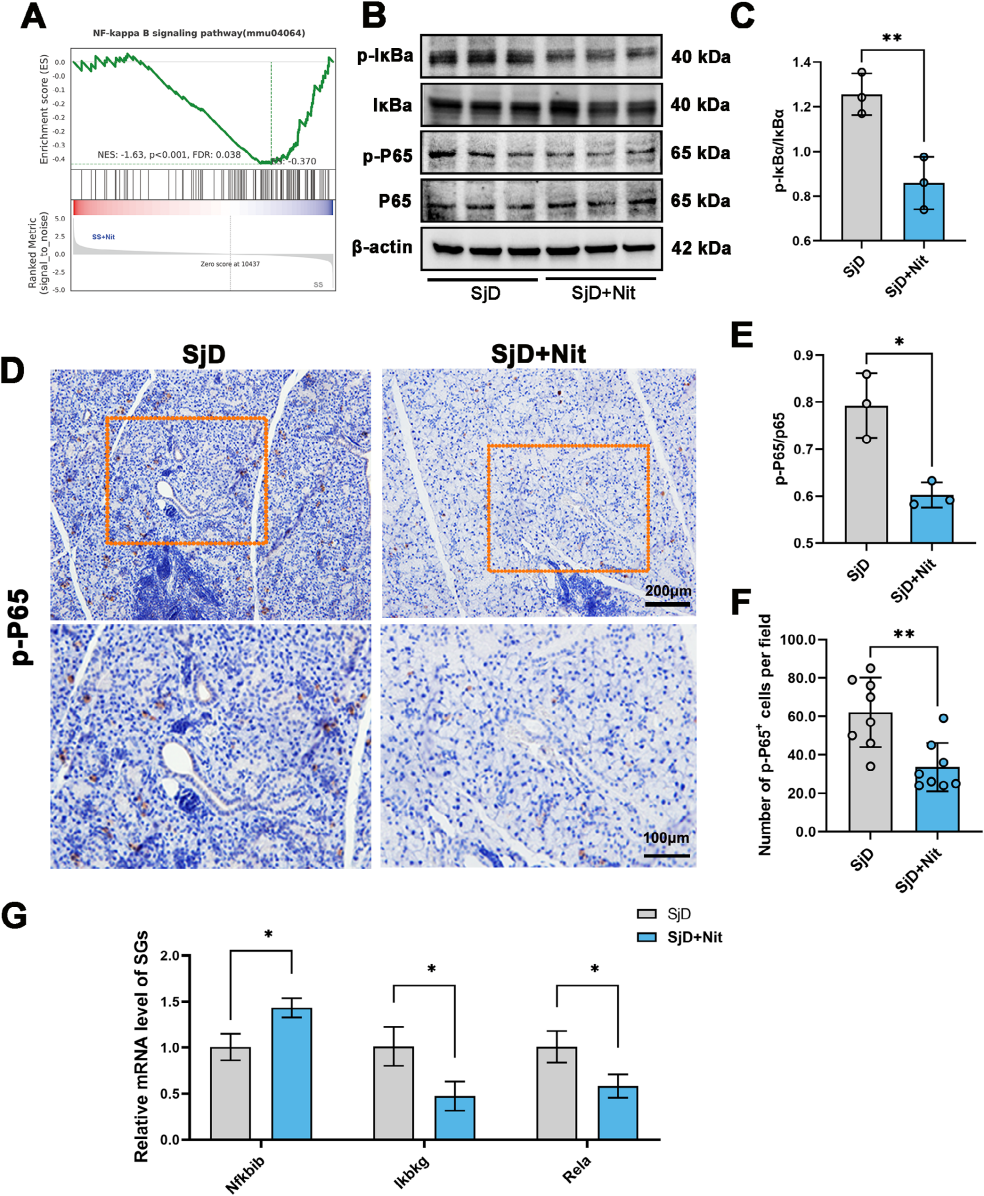
Nitrate‐induced improvement in SG function is associated with suppression of the NF‐κB pathway. (A) GSEA showed that NF‐κB signaling pathway in the SjD + Nit group was downregulated (*p* < 0.001). (B, C, E) Western blotting found that the ratio of p‐IκBα/IκBα and p‐P65/p65 in the SjD + Nit group was decreased compared with SjD group (*n* = 3, *p < 0.05, **p < 0.01). (D, F) IHC staining of p‐P65 and quantitative analysis showed that in the SjD + Nit group, the expression of p‐P65 was significantly lower than in the SjD group (*n* = 8, scale bar: 100 μm and 200 μm, **p < 0.01). (G) RNA level of *Nfκbib* were increased in SjD + Nit Group. *Ikbkg, Rela* were decreased in SjD + Nit Group (*n* = 3, *p < 0.05). Student's t‐test and ANOVA test was performed to compare difference between two groups with normal distribution.

NF‐κB, a prototypical pro‐inflammatory signaling pathway, regulates various pro‐inflammatory genes and plays vital roles in multiple inflammatory responses (Yu et al. 2020). Gene set enrichment analysis indicated that the NF‐κB signaling pathway was downregulated in the SjD + Nit group (*p* < 0.001; Figure 5A). The expression of p‐P65, P65, IκBα, and p‐IκBα in the SGs was detected, where the ratio of p‐P65/p65 and p‐IκBα/IκBα in the SjD + Nit group was significantly decreased compared with SjD group (*p < 0.05, **p < 0.01; Figure 5B,C,E). IHC staining showed that nitrate significantly decreased the expression of p‐P65 (**p < 0.01; Figure 5D,F). Similarly, The RNA level in the SGs were measured by qRT‐PCR. The mRNA expression *Nfκbib* were increased, and *Ikbkg* and *Rela* were decreased in SjD + Nit group (*p < 0.05; Figure 5G). Overall, nitrate treatment was associated with improved SG function, Th cell balance, and NF‐κB pathway activation.

## 4 Discussion

Sjögren's disease, a chronic systemic autoimmune disease of high prevalence, affects the exocrine glands and causes systemic autoimmune manifestations (Mavragani and Moutsopoulos 2014; Thalayasingam et al. 2021; Tian et al. 2021). Characteristics of SjD include lymphocyte infiltration of SGs, an imbalance in Th cells, and pro‐inflammatory cytokine production (Verstappen et al. 2021; Zhan et al. 2023). Current clinical treatments for SjD are mostly symptomatic, improving sicca symptoms (Fox et al. 2021). However, the preventive effect of nitrate on SjD has not been validated, particularly its ability to modulate immune cells, which requires further investigation. An optimal preventive treatment for SjD is yet to be established. Nitrate can inhibit inflammation, modulate the immune response, and re‐establish SG functions (Feng et al. 2021; Pan, Gu, et al. 2023; Pan, Hu, et al. 2023). The preventive effect of nitrate on SjD remains unclear. In this study, we demonstrate that nitrate is associated with maintaining salivary flow, revitalizing impaired SG function, rebalance of the Th cell ratio, and suppression of the NF‐κB pathway. Our study identifies the preventive potential of nitrate in the context of SG damage associated with SjD and some molecular and cellular changes associated with this glandular enhancement.

Preventive strategies for the onset or early stages of SjD have not been reported. Nitrate concentration and reabsorption by SGs (Qin et al. 2012) are significantly related to SjD dysfunction (Xia et al. 2003a, 2003b). Similarly, we found that salivary nitrate concentrations gradually decreased as SjD progressed. Nitrate administration modulates inflammation and suppresses oxidative stress (Chang et al. 2019). Additionally, nitrate shows great potential in preventing irradiation‐induced SG damage by improving mitochondrial function, suppressing pyroptosis (Li, An, et al. 2021; Li, Jin, et al. 2021), maintaining cell proliferation, and inhibiting apoptosis (Feng et al. 2021; Pan, Gu, et al. 2023; Pan, Hu, et al. 2023). In this study, nitrate pretreatment restored the SG function, resulting in increased salivary flow rate and decreased infiltration of lymphocytes. Although nitrate is crucial in SGs function, its underlying mechanism requires further investigation.

Based on our RNA sequencing results, nitrate pretreatment could downregulate the ER‐stress‐related pathways, while upregulating mucin O‐glycosylation, O‐linked glycosylation, and N‐glycosylation signaling pathway. Endoplasmic reticulum (ER) is the primary intracellular compartment responsible for protein biosynthesis and folding. ER stress potentially triggers autoimmunity (Stergios et al. 2019). ER stress is a key driver of acinar cell apoptosis and immune dysregulation in SjD. Chronic inflammation induces protein misfolding overload in salivary gland epithelial cells, as evidenced by aberrant MUC1 accumulation in the ER of SjD patients through TNF‐α/IFN‐γ‐mediated NF‐κB activation (González et al. 2019). In SjD, chronic ER stress has been implicated in acinar cell apoptosis and immune activation, exacerbating glandular dysfunction (Skopouli and Katsiougiannis 2018). Nitrate significantly reduces the expression of *ATF6*, *Ern1*, *Xbp1*, and *Ddit3*, suggesting alleviation of ER stress. This suggests that nitrate may mitigate ER stress‐induced glandular dysfunction, potentially disrupting the inflammatory loop between cytokine overproduction and mucin misfolding observed in SjD pathogenesis. Secreted proteins are folded and matured in the ER lumen and Golgi apparatus. N‐glycosylation is the most common and versatile processing system of proteins from the secretory route, playing important roles in protein folding, solubility, quality control, stability, intracellular destination, and function of most glycoproteins (Aebi 2013). Dysregulated O‐glycosylation affects mucins, potentially impairing salivary rheology and immune interactions, exacerbating oral dryness and microbial dysbiosis (Kamounah et al. 2024). Radiotherapy‐induced hyposalivation in head and neck cancer patients is associated with dysregulated O‐glycosylation, manifested as decreased MUC5B sulfation and loss of MUC7, which collectively contribute to sticky saliva and oral dryness (Almhöjd et al. 2021). The extent of O‐glycosylation abnormalities correlates with the degree of salivary hypofunction, suggesting a direct link between impaired glycan processing and glandular dysfunction in SjD (Gallo et al. 2019). Mechanistically, emerging evidence suggests that aberrant B cell receptor glycosylation in SjD may foster autoreactive B cell survival through impaired signal regulation, thereby driving the production of pathogenic autoantibodies including anti‐Ro/SSA and anti‐La/SSB. These insights position glycosylation pathway modulation as a promising therapeutic target for SjD (Morel et al. 2022). The upregulation of *C1galt1*, *St6galnac2*, and *B3gnt6* indicates increased O‐glycosylation, while the elevation of *Stt3a*, *Stt3b*, and *Dpagt1* suggests enhanced N‐glycosylation, both of which may contribute to the stabilization of mucins and improved glandular function.

Infiltrating lymphocytes in SGs include T cells, B cells, dendritic cells (DCs), macrophages, and other immune cells (Zhan et al. 2023). Among these, CD4^+^ T cells dominate the infiltrating T lymphocytes (Campos et al. 2016). The secreted cytokines stimulate excessive activation of B cells, leading to the production of autoantibodies and impaired SGs function (Zhou et al. 2024). Different CD4^+^ T cell subsets contribute to SjD pathogenesis, particularly Th1 and Th17 cells, which are essential in the initial stages of SjD (Verstappen et al. 2018). Th1 cells predominantly produce pro‐inflammatory cytokines such as IFN‐γ and IL‐2, which are higher in SGs of patients with SjD than non‐Sjögren's disease (nSjD)‐sicca (van Woerkom et al. 2005). Th2 cells inhibit Th1 responses by producing anti‐inflammatory cytokines, such as IL‐4, IL‐10, and IL‐13, indicating a balance between Th1 and Th2 cells (Meng et al. 2024). The early stages of SjD are primarily characterized by Th1 cell polarization. Increased levels of Th2 cells have been found in peripheral blood and SGs, with a balance shift towards Th1 cells correlating with disease severity. Our study found that the level of IFN‐γ significantly decreased in the peripheral blood, and the ratio of Th1/Th2 cells was regulated, indicting the anti‐inflammation and immune modulation effect of nitrate.

Th17 cells in the SGs produce IL‐17 and IL‐22, which also contribute to the pathogenesis of SjD (Zhan et al. 2023). Th17 cells can develop towards Th17.1 cells, co‐expressing IL‐17 and IFN‐γ, or towards Th1 cells producing IFN‐γ without IL‐17, supporting chronic inflammation and B cell activation in patients with SjD (Verstappen et al. 2018). In our study, flow cytometry analysis did not detect Th17.1 cells in the submandibular glands, which may account for the limited number of immune cells in the SGs. Both Treg and Th17 cells can be induced by TGF‐β. In the absence of IL‐6, TGF‐β promotes differentiation of Treg, whereas the combination of TGF‐β and IL‐6 drives Th17 differentiation. We found that after pretreatment of nitrate, the expression of TGF‐β significantly increased and *Il6st* was significantly decreased, indicating the shift towards Treg differentiation. The function of Treg cells in patients remains unclear, given that both Th17 and Treg subsets are equally increased in the peripheral blood and minor SGs of patients with SjD (Christodoulou et al. 2008; Katsifis et al. 2009). Several mouse models of SjD have shown an imbalance between Th17 and Treg cells, which correlates with disease severity (Verstappen et al. 2018). Our group has explored strategies to generate SSA/Ro‐antigen specific Tregs in vivo, which can suppress IFN‐γ production of CD4^+^ T cells and preserved SG function, indicting the essential role of Treg during the treatment (Xu et al. 2022). In this study, we found that pretreatment of nitrate modulated the local and peripheral Th17/Treg balance and significantly increased TGF‐β expression, aligning with previous findings regarding the polarization and activation of the Th17/Treg (Hu et al. 2020).

Additionally, after pretreatment with nitrate, T cell‐related genes, including *Il7r, Il6st*, and *Il2rg*, significantly decreased, indicating a possible action of nitrate. Increased numbers of IL‐7R^+^ cells were found in the SGs of patients with SjD compared with patients with sicca (Bikker et al. 2012). IL‐7 correlates with T cell infiltration and contributes to IFN‐γ–mediated Th1 cells, driving the plasticity of Th17 cells to IFN‐γ single or double‐producing Th17.1 cells (Arbelaez et al. 2015). Chemokines such as CXCL10 are related to the recruitment and migration of T cells (Liao et al. 2024) and were found to decrease following nitrate pretreatment in this study. This complex cytokine interaction network is related to the occurrence and development of SjD and may be a preventive target for nitrate.

Furthermore, the NF‐κB signaling pathway activates multiple inflammatory downstream targets, including pro‐inflammatory cytokines. Salivary epithelial cells chronically secrete IL‐6 and BAFF, which sustain lymphocyte infiltration, thereby perpetuating a pro‐inflammatory microenvironment (Manoussakis and Kapsogeorgou 2007). In SjD, both immune cells and salivary gland epithelial cells contribute to NF‐κB activation, forming a feed‐forward loop that exacerbates glandular dysfunction (Sisto et al. 2020). IκBα‐mediated feedback regulation of NF‐κB is crucial in regulating T cell development and function during SjD development (Peng et al. 2010). NF‐κB modulation in antigen‐presenting cells affects Th17 cell differentiation (Park et al. 2014). Cytokines secreted by Th1/Th17 cells, such as IFN‐γ, TNF‐α, and IL6, activate NF‐κB signaling in the progression of SjD (Yu et al. 2020). However, the correlation of the NF‐κB pathway and Th cell imbalance remains unclear. Notably, nitrate has been shown to suppress the NF‐κB pathway and modulate Th cells of SjD in this study. A limitation is that the sequence of T cells and NF‐κB signaling pathway regulated by nitrate has not been clarified. This complex relationship requires further investigation to explore the key mediators of pretreatment of nitrate.

Overall, nitrate pretreatment effectively prevents lymphocyte infiltration progression and preserves the impaired salivary function in NOD/LtJ mice by modulating Th cell balance and suppressing the NF‐κB signaling pathway. The complex underlying mechanism by which nitrate rebalances the Th cells and suppresses NF‐κB signaling remains unclear, and the crucial role of IFN‐γ, IL‐7, and other chemokines in the migration and polarization of Th cells needs further research. As a safe and cost‐effective additive sourced from vegetables, nitrate may serve as a potential preventive treatment for patients with SjD and holds promise for future clinical applications.

## Author Contributions

**Conglin Du:** methodology, data curation, investigation, writing – original draft. **Zi Yang:** methodology, data curation, investigation, funding acquisition, writing – original draft, validation. **Yang Yang:** investigation, data curation. **Chunmei Zhang:** visualization, writing – review and editing. **Hideaki Kagami:** writing – review and editing, conceptualization. **Xianqi Li:** writing – review and editing, conceptualization. **Liang Hu:** conceptualization, writing – review and editing, funding acquisition, supervision, investigation. **Junji Xu:** conceptualization, supervision, visualization, writing – review and editing. **Jian Zhou:** conceptualization, methodology, supervision, writing – review and editing, funding acquisition.

## Ethics Statement

This study was reviewed and approved by the Animal Care and Use Committee of the Capital Medical University (Code: AEEI‐2017‐116).

## Conflicts of Interest

The authors declare no conflicts of interest.

## Supporting information


**Figure S1.** Changes of salivary gland in NOD/LtJ mice from 6 to 16 weeks. (A, B) The lymphocyte infiltration area increased in NOD/LtJ mice submandibular gland. (C) The salivary flow rate gradually reduced in NOD/LtJ mice. (D)The nitrate concentration in saliva variation tendency in NOD/LtJ mice. *n* = 3. Pearson correlation coefficient was used to assess the linear relationship.

**Figure S2.** qRT‐PCR of submandibular glands in SjD and SjD + Nit group. (A) Gene related to immune and inflammation, (B) *Slc17a5* expression, (C) submandibular function, (D) Th cell, (E) p75NTR pathway, (F) O‐linked glycosylation, N‐glycosylation, (G) chemokine signaling and (H) ER stress changes in SGs. *n* = 3, * *p* < 0.05, ** *p* < 0.01, *** *p* < 0.001. Student's t‐test was performed to compare difference between two groups with normal distribution.

**Table S1.** Primers for qRT‐PCR.

## Data Availability

The datasets analyzed during the current study are available in the National Center for Biotechnology Information with the primary accession code PRJNA1161122.

## References

[odi70004-bib-0001] Aebi, M. 2013. “N‐Linked Protein Glycosylation in the ER.” Biochimica et Biophysica Acta 1833, no. 11: 2430–2437. 10.1016/j.bbamcr.2013.04.001. 23583305

[odi70004-bib-0002] Almhöjd, U. , H. Cevik‐Aras , N. Karlsson , J. Chuncheng , and A. Almståhl . 2021. “Stimulated Saliva Composition in Patients With Cancer of the Head and Neck Region.” BMC Oral Health 21, no. 1: 509. 10.1186/s12903-021-01872-x. 34627217 PMC8501675

[odi70004-bib-0003] Arbelaez, C. A. , S. Glatigny , R. Duhen , G. Eberl , M. Oukka , and E. Bettelli . 2015. “IL‐7/IL‐7 Receptor Signaling Differentially Affects Effector CD4^+^ T Cell Subsets Involved in Experimental Autoimmune Encephalomyelitis.” Journal of Immunology 195, no. 5: 1974–1983. 10.4049/jimmunol.1403135. PMC4546887 26223651

[odi70004-bib-0004] Bikker, A. , A. A. Kruize , M. Wenting , et al. 2012. “Increased Interleukin (IL)‐7Rα Expression in Salivary Glands of Patients With Primary Sjogren's Syndrome Is Restricted to T Cells and Correlates With IL‐7 Expression, Lymphocyte Numbers and Activity.” Annals of the Rheumatic Diseases 71, no. 6: 1027–1033. 10.1136/annrheumdis-2011-200744. 22312161

[odi70004-bib-0005] Campos, J. , M. R. Hillen , and F. Barone . 2016. “Salivary Gland Pathology in Sjögren's Syndrome.” Rheumatic Diseases Clinics of North America 42, no. 3: 473–483. 10.1016/j.rdc.2016.03.006. 27431349

[odi70004-bib-0007] Chang, S. , L. Hu , Y. Xu , et al. 2019. “Inorganic Nitrate Alleviates Total Body Irradiation‐Induced Systemic Damage by Decreasing Reactive Oxygen Species Levels.” International Journal of Radiation Oncology, Biology, Physics 103, no. 4: 945–957. 10.1016/j.ijrobp.2018.11.021. 30458235

[odi70004-bib-0008] Christodoulou, M. I. , E. K. Kapsogeorgou , and H. M. Moutsopoulos . 2010. “Characteristics of the Minor Salivary Gland Infiltrates in Sjögren's Syndrome.” Journal of Autoimmunity 34, no. 4: 400–407. 10.1016/j.jaut.2009.10.004. 19889514

[odi70004-bib-0009] Christodoulou, M. I. , E. K. Kapsogeorgou , N. M. Moutsopoulos , and H. M. Moutsopoulos . 2008. “Foxp3^+^ T‐Regulatory Cells in Sjogren's Syndrome: Correlation With the Grade of the Autoimmune Lesion and Certain Adverse Prognostic Factors.” American Journal of Pathology 173, no. 5: 1389–1396. 10.2353/ajpath.2008.080246. 18818377 PMC2570129

[odi70004-bib-0010] Feng, X. , Z. Wu , J. Xu , et al. 2021. “Dietary Nitrate Supplementation Prevents Radiotherapy‐Induced Xerostomia.” eLife 10: e70710. 10.7554/eLife.70710. 34581269 PMC8563005

[odi70004-bib-0011] Fox, R. I. , C. M. Fox , J. E. Gottenberg , and T. Dörner . 2021. “Treatment of Sjögren's Syndrome: Current Therapy and Future Directions.” Rheumatology 60, no. 5: 2066–2074. 10.1093/rheumatology/kez142. 31034046

[odi70004-bib-0012] Gallo, A. , S. Vella , F. Tuzzolino , et al. 2019. “MicroRNA‐Mediated Regulation of Mucin‐Type O‐Glycosylation Pathway: A Putative Mechanism of Salivary Gland Dysfunction in Sjögren Syndrome.” Journal of Rheumatology 46, no. 11: 1485–1494. 10.3899/jrheum.180549. 30824638

[odi70004-bib-0013] Hu, L. , L. Jin , D. Xia , et al. 2020. “Nitrate Ameliorates Dextran Sodium Sulfate‐Induced Colitis by Regulating the Homeostasis of the Intestinal Microbiota.” Free Radical Biology and Medicine 152: 609–621. 10.1016/j.freeradbiomed.2019.12.002. 31811920

[odi70004-bib-0014] Iizuka, M. , H. Tsuboi , N. Matsuo , et al. 2015. “A Crucial Role of RORγt in the Development of Spontaneous Sialadenitis‐Like Sjögren's Syndrome.” Journal of Immunology 194, no. 1: 56–67. 10.4049/jimmunol.1401118. 25411202

[odi70004-bib-0015] Kamounah, S. , K. A. Thomsson , C. E. Sørensen , E. P. Bennett , N. G. Karlsson , and A. M. L. Pedersen . 2024. “Altered O‐Glycans in Stimulated Whole Saliva From Patients With Primary Sjögren's Syndrome and Non‐pSS Sicca.” Scientific Reports 14, no. 1: 29377. 10.1038/s41598-024-79473-1. 39592783 PMC11599585

[odi70004-bib-0016] Katsifis, G. E. , S. Rekka , N. M. Moutsopoulos , S. Pillemer , and S. M. Wahl . 2009. “Systemic and Local Interleukin‐17 and Linked Cytokines Associated With Sjögren's Syndrome Immunopathogenesis.” American Journal of Pathology 175, no. 3: 1167–1177. 10.2353/ajpath.2009.090319. 19700754 PMC2731135

[odi70004-bib-0018] Li, S. , W. An , B. Wang , et al. 2021. “Inorganic Nitrate Alleviates Irradiation‐Induced Salivary Gland Damage by Inhibiting Pyroptosis.” Free Radical Biology & Medicine 175: 130–140. 10.1016/j.freeradbiomed.2021.08.227. 34454049

[odi70004-bib-0019] Li, S. , H. Jin , G. Sun , et al. 2021. “Dietary Inorganic Nitrate Protects Hepatic Ischemia‐Reperfusion Injury Through NRF2‐Mediated Antioxidative Stress.” Frontiers in Pharmacology 12: 634115. 10.3389/fphar.2021.634115. 34163351 PMC8215696

[odi70004-bib-0020] Liao, J. , X. Yu , Z. Huang , et al. 2024. “Chemokines and Lymphocyte Homing in Sjögren's Syndrome.” Frontiers in Immunology 15: 1345381. 10.3389/fimmu.2024.1345381. 38736890 PMC11082322

[odi70004-bib-0021] Ma, L. , L. Hu , X. Feng , and S. Wang . 2018. “Nitrate and Nitrite in Health and Disease.” Aging and Disease 9, no. 5: 938–945. 10.14336/ad.2017.1207. 30271668 PMC6147587

[odi70004-bib-0022] Manoussakis, M. N. , and E. K. Kapsogeorgou . 2007. “The Role of Epithelial Cells in the Pathogenesis of Sjögren's Syndrome.” Clinical Reviews in Allergy and Immunology 32, no. 3: 225–230. 10.1007/s12016-007-8007-4. 17992589

[odi70004-bib-0023] Mavragani, C. P. , and H. M. Moutsopoulos . 2014. “Sjogren's Syndrome.” Annual Review of Pathology 9: 273–285. 10.1146/annurev-pathol-012513-104728. 24050623

[odi70004-bib-0024] Meng, Q. , J. Ma , J. Cui , Y. Gu , and Y. Shan . 2024. “Subpopulation Dynamics of T and B Lymphocytes in Sjögren's Syndrome: Implications for Disease Activity and Treatment.” Frontiers in Immunology 15: 1468469. 10.3389/fimmu.2024.1468469. 39290700 PMC11405198

[odi70004-bib-0025] Morel, M. , P. Pochard , W. Echchih , et al. 2022. “Abnormal B Cell Glycosylation in Autoimmunity: A New Potential Treatment Strategy.” Frontiers in Immunology 13: 975963. 10.3389/fimmu.2022.975963. 36091064 PMC9453492

[odi70004-bib-0027] Pan, W. , J. Gu , S. Xu , et al. 2023. “Dietary Nitrate Improves Jaw Bone Remodelling in Zoledronate‐Treated Mice.” Cell Proliferation 56, no. 7: e13395. 10.1111/cpr.13395. 36810909 PMC10334281

[odi70004-bib-0028] Pan, W. , G. Hu , S. Li , et al. 2023. “Nanonitrator: Novel Enhancer of Inorganic Nitrate's Protective Effects, Predicated on Swarm Learning Approach.” Science Bulletin (Beijing) 68, no. 8: 838–850. 10.1016/j.scib.2023.03.043. 37029030

[odi70004-bib-0029] Park, S. H. , G. Cho , and S. G. Park . 2014. “NF‐κB Activation in T Helper 17 Cell Differentiation.” Immune Network 14, no. 1: 14–20. 10.4110/in.2014.14.1.14. 24605076 PMC3942503

[odi70004-bib-0030] Peng, B. , J. Ling , A. J. Lee , et al. 2010. “Defective Feedback Regulation of NF‐kappaB Underlies Sjogren's Syndrome in Mice With Mutated kappaB Enhancers of the IkappaBalpha Promoter.” Proceedings of the National Academy of Sciences of the United States of America 107, no. 34: 15193–15198. 10.1073/pnas.1005533107. 20696914 PMC2930541

[odi70004-bib-0031] Psianou, K. , I. Panagoulias , A. D. Papanastasiou , et al. 2018. “Clinical and Immunological Parameters of Sjögren's Syndrome.” Autoimmunity Reviews 17, no. 10: 1053–1064. 10.1016/j.autrev.2018.05.005. 30103041

[odi70004-bib-0032] Qin, B. , J. Wang , Z. Yang , et al. 2015. “Epidemiology of Primary Sjögren's Syndrome: A Systematic Review and Meta‐Analysis.” Annals of the Rheumatic Diseases 74, no. 11: 1983–1989. 10.1136/annrheumdis-2014-205375. 24938285

[odi70004-bib-0033] Qin, L. , X. Liu , Q. Sun , et al. 2012. “Sialin (SLC17A5) Functions as a Nitrate Transporter in the Plasma Membrane.” Proceedings of the National Academy of Sciences of the United States of America 109, no. 33: 13434–13439. 10.1073/pnas.1116633109. 22778404 PMC3421170

[odi70004-bib-0034] Qu, X. , Z. Wu , B. Pang , L. Jin , L. Qin , and S. Wang . 2016. “From Nitrate to Nitric Oxide: The Role of Salivary Glands and Oral Bacteria.” Journal of Dental Research 95, no. 13: 1452–1456. 10.1177/0022034516673019. 27872324

[odi70004-bib-0035] Sebastian, A. , A. Szachowicz , and P. Wiland . 2019. “Classification Criteria for Secondary Sjögren's Syndrome. Current State of Knowledge.” Rheumatology 57, no. 5: 277–280. 10.5114/reum.2019.89520. 31844340 PMC6911247

[odi70004-bib-0036] Sisto, M. , D. Ribatti , and S. Lisi . 2020. “Understanding the Complexity of Sjögren's Syndrome: Remarkable Progress in Elucidating NF‐κB Mechanisms.” Journal of Clinical Medicine 9, no. 9: 2821. 10.3390/jcm9092821. 32878252 PMC7563658

[odi70004-bib-0037] Skopouli, F. N. , and S. Katsiougiannis . 2018. “How Stress Contributes to Autoimmunity‐Lessons From Sjögren's Syndrome.” FEBS Letters 2018, no. 592: 5–14. 10.1002/1873-3468.12933. 29223133

[odi70004-bib-0038] Thalayasingam, N. , K. Baldwin , C. Judd , and W. F. Ng . 2021. “New Developments in Sjogren's Syndrome.” Rheumatology (Oxford, England) 60, no. Supplement_6: vi53–vi61. 10.1093/rheumatology/keab466. 34951923 PMC8709567

[odi70004-bib-0039] Tian, Y. , H. Yang , N. Liu , Y. Li , J. Chen , and Y. Yang . 2021. “Advances in Pathogenesis of Sjögren's Syndrome.” Journal of Immunology Research 2021: 5928232. 10.1155/2021/5928232. 34660815 PMC8516582

[odi70004-bib-0040] van Woerkom, J. M. , A. A. Kruize , M. J. Wenting‐Van Wijk , et al. 2005. “Salivary Gland and Peripheral Blood T Helper 1 and 2 Cell Activity in Sjögren's Syndrome Compared With Non‐Sjögren's Sicca Syndrome.” Annals of the Rheumatic Diseases 64, no. 10: 1474–1479. 10.1136/ard.2004.031781. 15817659 PMC1755251

[odi70004-bib-0041] Verstappen, G. M. , O. B. Corneth , H. Bootsma , and F. G. Kroese . 2018. “Th17 Cells in Primary Sjögren's Syndrome: Pathogenicity and Plasticity.” Journal of Autoimmunity 87: 16–25. 10.1016/j.jaut.2017.11.003. 29191572

[odi70004-bib-0042] Verstappen, G. M. , F. G. M. Kroese , and H. Bootsma . 2021. “T Cells in Primary Sjögren's Syndrome: Targets for Early Intervention.” Rheumatology (Oxford, England) 60, no. 7: 3088–3098. 10.1093/rheumatology/kez004. 30770920 PMC8516500

[odi70004-bib-0043] Wang, W. , L. Hu , S. Chang , et al. 2020. “Total Body Irradiation‐Induced Colon Damage Is Prevented by Nitrate‐Mediated Suppression of Oxidative Stress and Homeostasis of the Gut Microbiome.” Nitric Oxide 102: 1–11. 10.1016/j.niox.2020.05.002. 32470598

[odi70004-bib-0044] Weitzberg, E. , and J. O. Lundberg . 2013. “Novel Aspects of Dietary Nitrate and Human Health.” Annual Review of Nutrition 33: 129–159. 10.1146/annurev-nutr-071812-161159. 23642194

[odi70004-bib-0045] Xia, D. , D. Deng , and S. Wang . 2003a. “Alterations of Nitrate and Nitrite Content in Saliva, Serum, and Urine in Patients With Salivary Dysfunction.” Journal of Oral Pathology & Medicine 32, no. 2: 95–99. 10.1034/j.1600-0714.2003.00109.x. 12542832

[odi70004-bib-0046] Xia, D. , D. Deng , and S. Wang . 2003b. “Destruction of Parotid Glands Affects Nitrate and Nitrite Metabolism.” Journal of Dental Research 82, no. 2: 101–105. 10.1177/154405910308200205. 12562881

[odi70004-bib-0047] Xu, J. , O. Liu , D. Wang , et al. 2022. “In Vivo Generation of SSA/Ro Antigen‐Specific Regulatory T Cells Improves Experimental Sjögren's Syndrome in Mice.” Arthritis & Rhematology 74, no. 10: 1699–1705. 10.1002/art.42244. PMC9811988 35606923

[odi70004-bib-0048] Xu, Y. , B. Pang , L. Hu , et al. 2018. “Dietary Nitrate Protects Submandibular Gland From Hyposalivation in Ovariectomized Rats via Suppressing Cell Apoptosis.” Biochemical and Biophysical Research Communications 497, no. 1: 272–278. 10.1016/j.bbrc.2018.02.068. 29432741

[odi70004-bib-0049] Yu, H. , L. Lin , Z. Zhang , H. Zhang , and H. Hu . 2020. “Targeting NF‐κB Pathway for the Therapy of Diseases: Mechanism and Clinical Study.” Signal Transduction and Targeted Therapy 5, no. 1: 209. 10.1038/s41392-020-00312-6. 32958760 PMC7506548

[odi70004-bib-0050] Zhan, Q. , J. Zhang , Y. Lin , W. Chen , X. Fan , and D. Zhang . 2023. “Pathogenesis and Treatment of Sjogren's Syndrome: Review and Update.” Frontiers in Immunology 14: 1127417. 10.3389/fimmu.2023.1127417. 36817420 PMC9932901

[odi70004-bib-0051] Zhou, J. , J. L. Pathak , T. Cao , et al. 2024. “CD4 T Cell‐Secreted IFN‐γ in Sjögren's Syndrome Induces Salivary Gland Epithelial Cell Ferroptosis.” Biochimica et Biophysica Acta – Molecular Basis of Disease 1870, no. 4: 167121. 10.1016/j.bbadis.2024.167121. 38471652

