# Supplementary figures and images for: Nitrate Prevents Sjögren's Disease by Modulating T Helper Cells via NF‐κB Pathway Suppression
Source: Oral Dis. 2025 Jun 16;31(12):3323–35. doi: 10.1111/odi.70004 (PMC12989053; doi:10.1111/odi.70004)

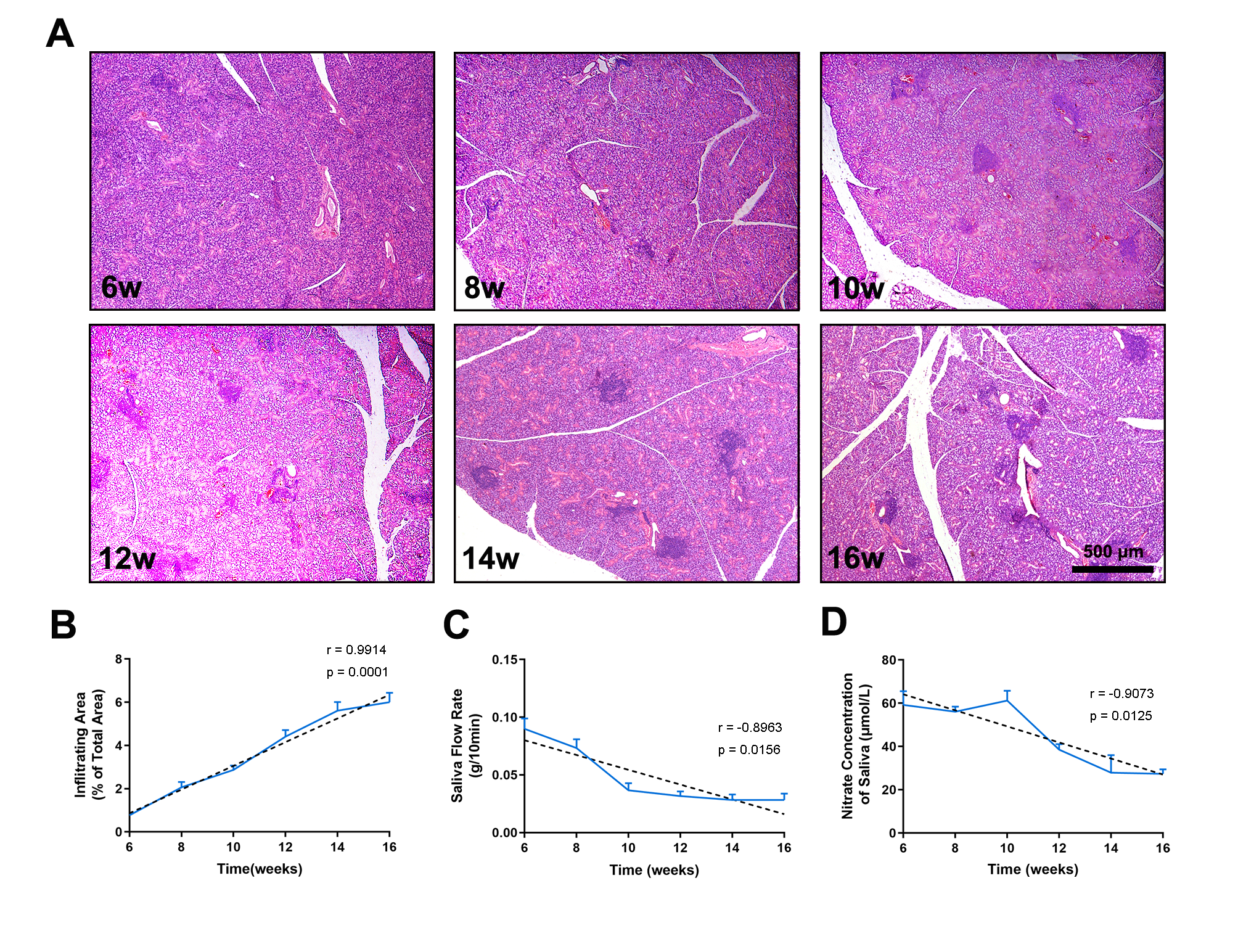

Supplement: Supplementary file 1 — Figure S1. Changes of salivary gland in NOD/LtJ mice from 6 to 16 weeks. (A, B) The lymphocyte infiltration area increased in NOD/LtJ mice submandibular gland. (C) The salivary flow rate gradually reduced in NOD/LtJ mice. (D)The nitrate concentration in saliva variation tendency in NOD/LtJ mice. n = 3. Pearson correlation coefficient was used to assess the linear relationship. [file ODI-31-3323-s003.zip › odi70004-sup-0001-FigureS1@Fig.S1_1.tif]

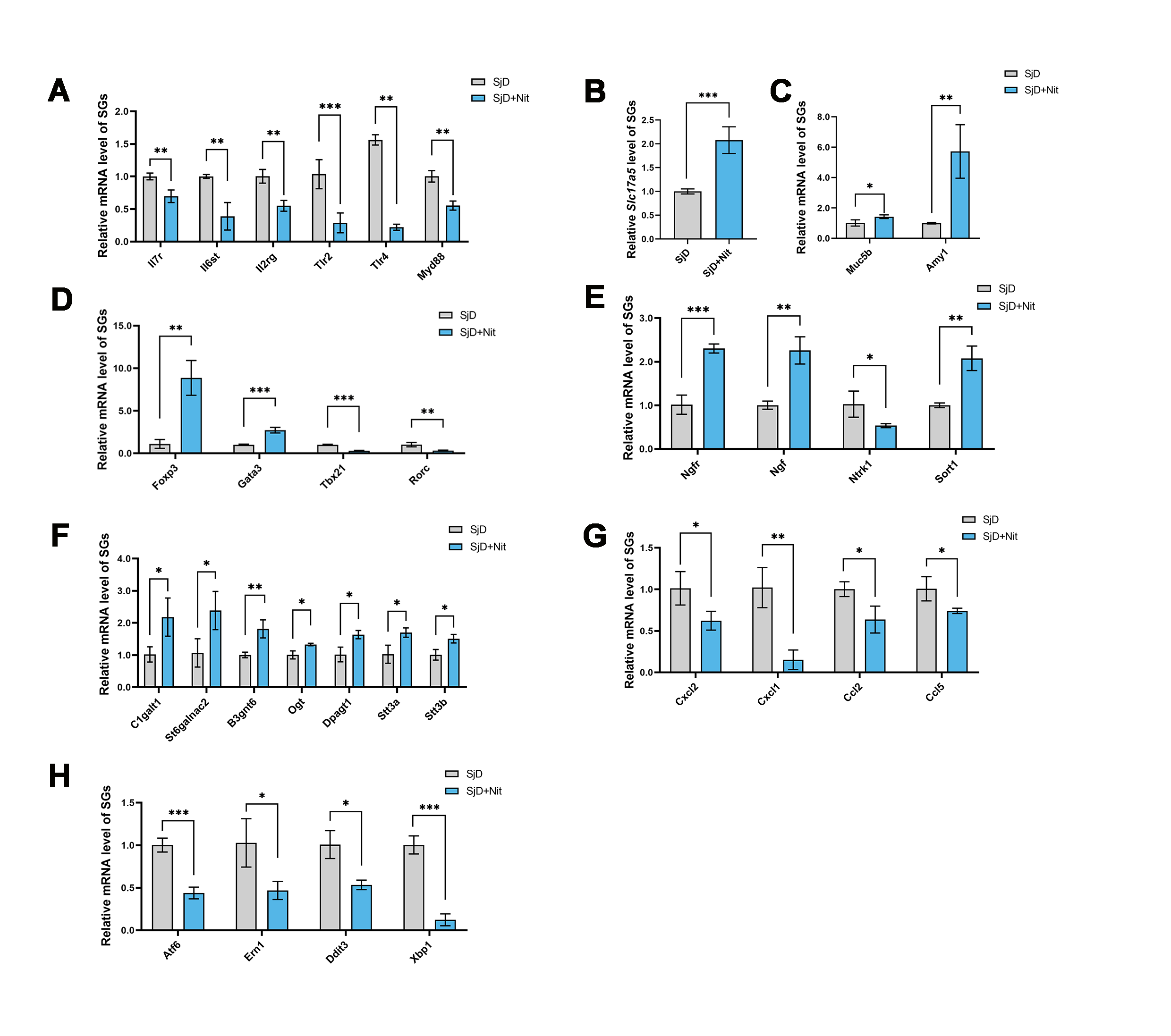

Supplement: Supplementary file 2 — Figure S2. qRT‐PCR of submandibular glands in SjD and SjD + Nit group. (A) Gene related to immune and inflammation, (B) Slc17a5 expression, (C) submandibular function, (D) Th cell, (E) p75NTR pathway, (F) O‐linked glycosylation, N‐glycosylation, (G) chemokine signaling and (H) ER stress changes in SGs. n = 3, * p < 0.05, ** p < 0.01, *** p < 0.001. Student's t‐test was performed to compare difference between two groups with normal distribution. [file ODI-31-3323-s002.zip › odi70004-sup-0003-FigureS2@FIG.S2_1.tif]
