# Supplementary material for: Nitrate Prevents Sjögren's Disease by Modulating T Helper Cells via NF‐κB Pathway Suppression
Source: Oral Dis. 2025 Jun 16;31(12):3323–35. doi: 10.1111/odi.70004 (PMC12989053; doi:10.1111/odi.70004)
Supplement: Supplementary file 3 — Table S1. Primers for qRT‐PCR. [file ODI-31-3323-s001.zip › odi70004-sup-0005-TableS1@Supplementary Table1_1.docx]

**Supplementary Table S1. Primers for qRT-PCR.**

| Rela F | AGGCTTCTGGGCCTTATGTG |
| --- | --- |
| Rela R | TGCTTCTCTCGCCAGGAATAC |
| Ikbkg F | AAGCACCCCTGGAAGAACC |
| Ikbkg R | CCTGCTCTGAAGGCAGATGTA |
| Nfkbib F | GCGGATGCCGATGAATGGT |
| Nfkbib R | TGACGTAGCCAAAGACTAAGGG |
| Gata3 F | CTCGGCCATTCGTACATGGAA |
| Gata3 R | GGATACCTCTGCACCGTAGC |
| Foxp3 F | CCCATCCCCAGGAGTCTTG |
| Foxp3 R | ACCATGACTAGGGGCACTGTA |
| Rorc F | GACCCACACCTCACAAATTGA |
| Rorc R | AGTAGGCCACATTACACTGCT |
| Tbx21 F | AGCAAGGACGGCGAATGTT |
| Tbx21 R | GGGTGGACATATAAGCGGTTC |
| Muc5b F | GCCGAGGCAAGTACCTGTC |
| Muc5b R | ACAGCCCTTATACCGCAAGAC |
| Il7r F | GCGGACGATCACTCCTTCTG |
| Il7r R | AGCCCCACATATTTGAAATTCCA |
| Il6st F | CCGTGTGGTTACATCTACCCT |
| Il6st R | CGTGGTTCTGTTGATGACAGTG |
| Il2rg F | CTCAGGCAACCAACCTCAC |
| Il2rg R | GCTGGACAACAAATGTCTGGTAG |
| Amy1 F | AACCCAAATAACAGGGACTTTCC |
| Amy1 R | GGTAGTTCTCGATACCTCCACTT |
| Tlr2 F | GCAAACGCTGTTCTGCTCAG |
| Tlr2 R | AGGCGTCTCCCTCTATTGTATT |
| Tlr4 F | ATGGCATGGCTTACACCACC |
| Tlr4 R | GAGGCCAATTTTGTCTCCACA |
| Ngfr F | CTAGGGGTGTCCTTTGGAGGT |
| Ngfr R | CAGGGTTCACACACGGTCT |
| Ngf F | CCAGTGAAATTAGGCTCCCTG |
| Ngf R | CCTTGGCAAAACCTTTATTGGG |
| Sort1 F | CCCGGACTTCATCGCCAAG |
| Sort1 R | AGGACGAGAATAACCCCAGTG |
| Ntrk1 F | GCCTAACCATCGTGAAGAGTG |
| Ntrk1 R | CCAACGCATTGGAGGACAGAT |
| Cxcl1 F | CTGGGATTCACCTCAAGAACATC |
| Cxcl1 R | CAGGGTCAAGGCAAGCCTC |
| Cxcl2 F | CCAACCACCAGGCTACAGG |
| Cxcl2 R | GCGTCACACTCAAGCTCTG |
| Slc17a5 F | TCTGCTCGGTACAACTTAGCG |
| Slc17a5 R | CCGTAAGGCATAGAGAACGAAGA |
| Aqp5 F | AGAAGGAGGTGTGTTCAGTTGC |
| Aqp5 R | GCCAGAGTAATGGCCGGAT |
| Ccl2 F | TTAAAAACCTGGATCGGAACCAA |
| Ccl2 R | GCATTAGCTTCAGATTTACGGGT |
| Ccl5 F | GCTGCTTTGCCTACCTCTCC |
| Ccl5 R | TCGAGTGACAAACACGACTGC |
| Myd88 F | TCATGTTCTCCATACCCTTGGT |
| Myd88 R | AAACTGCGAGTGGGGTCAG |
| ATF6 F | CGGTCCACAGACTCGTGTTC |
| ATF6 R | GCTGTCGCCATATAAGGAAAGG |
| Xbp1 F | AGCAGCAAGTGGTGGATTTG |
| Xbp1 R | GAGTTTTCTCCCGTAAAAGCTGA |
| Ogt F | GACGCAACCAAACTTTGCAGT |
| Ogt R | TCAAGGGTGACAGCCTTTTCA |
| C1galt1 F | ATGGCCTCTAAATCTTGGCTGA |
| C1galt1 R | AGCCTCTTCTCGCAACAAAATA |
| St6galnac2 F | CCTCATGCTGTACTCCTCGG |
| St6galnac2 R | CGGTGGTTTGGGGTCAAAGA |
| B3gnt6 F | AAGAGTCCCACGACACTGG |
| B3gnt6 R | GTAGCGCAGGAAGTCTTGGA |
| Dpagt1 F | CTCGCTGTTGGGATTCGTG |
| Dpagt1 R | GCTGAGCTTGTTGAGGTCCTG |
| Stt3a F | TGTCGATGGCTGCTGTGTTAT |
| Stt3a R | CAGAAACCGGGTAGTCCGAT |
| Stt3b F | ATCCACGAGTTCGACCCGT |
| Stt3b R | ACCATGCTCTTTCATCAAACCA |
| Ddit3 F | CTGGAAGCCTGGTATGAGGAT |
| Ddit3 R | CAGGGTCAAGAGTAGTGAAGGT |
